# Transport and Field Emission Properties of MoS_2_ Bilayers

**DOI:** 10.3390/nano8030151

**Published:** 2018-03-08

**Authors:** Francesca Urban, Maurizio Passacantando, Filippo Giubileo, Laura Iemmo, Antonio Di Bartolomeo

**Affiliations:** 1 Department of Physics “E.R. Caianiello”, University of Salerno, 84084 Fisciano, Italy; furban@unisa.it (F.U.); liemmo@unisa.it (L.I.); 2 CNR-SPIN Salerno, 84084 Fisciano, Italy; filippo.giubileo@spin.cnr.it; 3 Department of Physical and Chemical Sciences, University of L’Aquila, and CNR-SPIN L’Aquila, 67100 L’Aquila, Italy; maurizio.passacantando@aquila.infn.it

**Keywords:** Transition metal dichalcogenides, MoS_2_, field-effect transistor, field emission

## Abstract

We report the electrical characterization and field emission properties of ${\mathrm{MoS}}_{2}$ bilayers deposited on a ${\mathrm{SiO}}_{2}/\mathrm{Si}$ substrate. Current–voltage characteristics are measured in the back-gate transistor configuration, with Ti contacts patterned by electron beam lithography. We confirm the n-type character of as-grown ${\mathrm{MoS}}_{2}$ and we report normally-on field-effect transistors. Local characterization of field emission is performed inside a scanning electron microscope chamber with piezo-controlled tungsten tips working as the anode and the cathode. We demonstrate that an electric field of ~200 V/μm is able to extract current from the flat part of ${\mathrm{MoS}}_{2}$ bilayers, which can therefore be conveniently exploited for field emission applications even in low field enhancement configurations. We show that a Fowler–Nordheim model, modified to account for electron confinement in two-dimensional (2D) materials, fully describes the emission process.

## 1. Introduction

Over the past decade, graphene and graphene-like materials have attracted a lot of attention. Due to its two-dimensional (2D) nature and several extraordinary properties, such as high mobility and current carrying capability, chemical stability, and mechanical robustness, graphene in particular has been the most commonly chosen material for new electronic devices [1,2]. However, the absence of an intrinsic bandgap has hampered its application as a transistor channel [3,4] and has paved the way for the study of alternative 2D materials with semiconducting behavior, such as the transition metal dichalcogenides (TMDs) [5,6]. The TMD family, which comprises ${\mathrm{MX}}_{2}$ compounds where M is a transition metal (Mo, W, etc.) and X a chalcogen (S, Se, Te), is gaining popularity in scientific and engineering research. In particular, molybdenum disulfide (${\mathrm{MoS}}_{2}$) is intensively studied for its ease of fabrication and direct bandgap suitable for optoelectronic applications [7,8,9,10].

Similarly to graphene, ${\mathrm{MoS}}_{2}$ can be mechanically exfoliated from a bulk material and transferred onto a substrate [6]; however, single-crystal and large-scale flakes with a controlled number of layers are more easily produced by chemical vapor deposition (CVD) [11]. ${\mathrm{MoS}}_{2}$ presents a structure consisting of a hexagonal plane of Mo atoms sandwiched between two planes of S atoms. Each layer is bonded to another one by van der Waals interactions to form the bulk structure. ${\mathrm{MoS}}_{2}$ is considered a good candidate for electronic and optoelectronic applications because it offers control on the width of the energy bandgap through the number of layers [12], even though its mobility is typically only a few tens ${\mathrm{cm}}^{2}V^{-1}s^{-1}$ [8]. Indeed, multilayer ${\mathrm{MoS}}_{2}$ shows an indirect bandgap of 1.2 eV which increases with the decreasing number of layers, becoming direct and of 1.8–1.9 eV for monolayers. The presence of an energy gap enables ${\mathrm{MoS}}_{2}$ transistors with a high on/off ratio $(>10^{7}$) [13] and low subthreshold swing (below 70 mV/decade) [14]; moreover, a field effect mobility of about $200\;{\mathrm{cm}}^{2}V^{-1}s^{-1}$ has been experimentally achieved [15]. Additional features such as high photoresponsivity [10,16] and spin–orbit splitting [17] have been investigated, opening the route for applicability of ${\mathrm{MoS}}_{2}$ for optoelectronic and spintronic devices [10,18]. 

In this paper, we characterize the transport properties of bilayer ${\mathrm{MoS}}_{2}$ field-effect transistors (FET) in the back-gate configuration. Furthermore, taking advantage of contacted ${\mathrm{MoS}}_{2}$ flakes, we investigate their local field emission behavior. Compared with monolayers, bilayers are less affected by ambient exposure [19] and tend to form smaller Schottky barriers with metal contacts [20]; thus, they are more suitable for field emission applications.

Field emission (FE) is a quantum mechanical phenomenon in which electrons, extracted from a conductor or a semiconductor surface upon application of an intense electric field, flow in vacuum from a cathode to an anode. Classic field emission theory was developed by Fowler and Nordheim [21] for planar electrodes, but it is usually also applied to electrodes forming sharp tips [22]. Actually, tips with small radius of curvature enable enhancement of the local electric field, thereby requiring a reduced anode-to-cathode field for electron extraction [23]. Nanostructures, such as nanoparticles [24,25], nanowires [26], and nanotubes [23,27,28,29,30] or 2D materials [31,32], for their intrinsically sharp edges and high aspect ratio, are natural field emission sources. Indeed, semiconducting or metallic nanostructured materials have been considered for FE applications in vacuum electronics [33], flat panel displays [34], electron microscopy [35], X-ray tubes [36,37], etc.

To date, FE from ${\mathrm{MoS}}_{2}$ has been very poorly characterized. There are few works concerning field emission measurements on ${\mathrm{MoS}}_{2}$ nanostructures, like single- and multilayer flakes, nanoflowers, and films that are edge-terminated vertically aligned (ETVA) [38]. It has also been reported that ultra-thin ETVA-${\mathrm{MoS}}_{2}$ films present FE characteristics comparable to those of carbon-based structures [39,40,41]. 

Here, we measure FE current from the flat part of ${\mathrm{MoS}}_{2}$ bilayers at a turn-on field of 230 V/μm. Despite the fact that we operate under no field enhancement condition, we find a local electric field magnified by a factor of 10 at the cathode–anode distance d=75 nm and find that the field amplification factor increases linearly with d. Remarkably, we show that FE from ${\mathrm{MoS}}_{2}$ bilayers follows a modified Fowler–Nordheim (FN) model recently proposed to include the effect of the confinement in 2D materials [42]. We highlight that, to the best of our knowledge, field emission from ${\mathrm{MoS}}_{2}$ bilayers has not been reported before. Our study, demonstrating its suitability as a field emitter, aims to exploit ${\mathrm{MoS}}_{2}$ in vacuum electronics, thus extending its use as an electrode in heterojunctions and channel in field-effect transistors.

## 2. Materials and Methods

**Figure 1:**
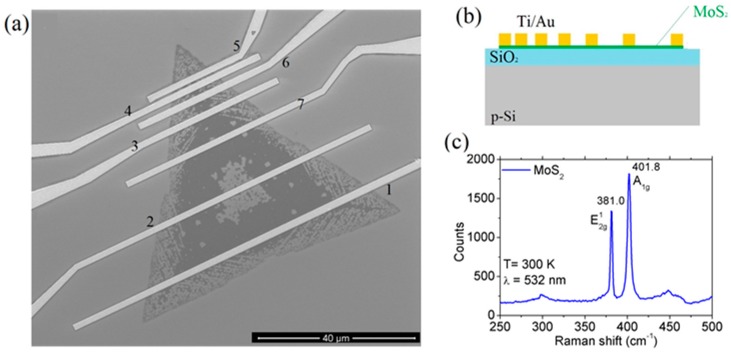
(**a**) SEM image of a Ti/Au contacted ${\mathrm{MoS}}_{2}$ flake; (**b**) Schematic cross section of the field-effect device; (**c**) Raman spectrum of the flake.

The ${\mathrm{MoS}}_{2}$ flakes were grown by CVD at 750 K (using S and ${\mathrm{MoO}}_{3}$ as precursors) on p-doped Si substrate covered by 300 nm of ${\mathrm{SiO}}_{2}$. The process yielded mainly bilayers and multilayers randomly distributed on the substrate. From scanning electron microscope (SEM, LEO 1530, Zeiss, Oberkochen, Germany) imaging, we often found traces of unreacted ${\mathrm{MoO}}_{3}$ precursor on the flake, as shown in Figure 1a, which displays a typical back-gated field-effect transistor with Ti/Au metal leads. Such residuals can affect the carrier mobility. A schematic of the device, consisting of a TLM structure (Transfer Length Method) with back gate, is reported in Figure 1b. 

We used the silicon substrate as a common back gate and metal leads, patterned by standard electron beam lithography and a lift-off process, as drain and source. The metal leads are made of Ti (20 nm) and Au (130 nm) deposited as contact and cover layers, respectively.

${\mathrm{MoS}}_{2}$ flakes were characterized by Raman spectroscopy before device fabrication in order to identify the number of layers. The Raman spectrum reported in Figure 1c shows two identifying peaks: $A_{1g}$ associated with the out-of-plane vibration of sulfur atoms and $E_{2g}^{1}$ resulting from the in-plane vibrations of Mo and S atoms [43]. The two peaks are separated by $21{\;\mathrm{cm}}^{-1}$, indicating bilayer flakes. Micro-Raman spectroscopy mapping evidenced uniform thickness across the whole flakes; in few cases, we observed thinning in localized regions. 

In the following, the transistor characterization refers to contacts 4 (drain) and 5 (source), as marked in Figure 1a, i.e., to the device with the shortest channel, which is the most interesting from an application perspective. The distance between the two contacts, i.e., the channel length, is L=0.57 μm, while the channel width is W≈11.4 μm. 

Electrical measurements were performed using a Keithley 4200 SCS (source measurement unit, Tektronix, Beaverton, OR, USA) connected to a Janis ST-500 (Janis Research Company, ST-500, Woburn, MA, USA) probe station at room temperature and pressure of ~3 mbar. 

Field emission measurements were carried out at a pressure $<10^{-6}\;\mathrm{mbar}$ in a SEM vacuum chamber endowed with two piezo-controlled tungsten tips (W-tips) with nanometric resolution.

Considering that the presence of impurities and precursor residues could alter the emission process from ${\mathrm{MoS}}_{2}$, we chose emitting areas that were cleaner and more homogeneous. We did not observe significant differences in these zones and we report only one dataset in the following. We also tried field emission from regions covered by ${\mathrm{MoO}}_{3}$, but we did not observe any signal. This is likely due to the 6.6 eV high work function of ${\mathrm{MoO}}_{3}$ [44]. 

## 3. Results

### 3.1. Transistor Characterization

**Figure 2:**
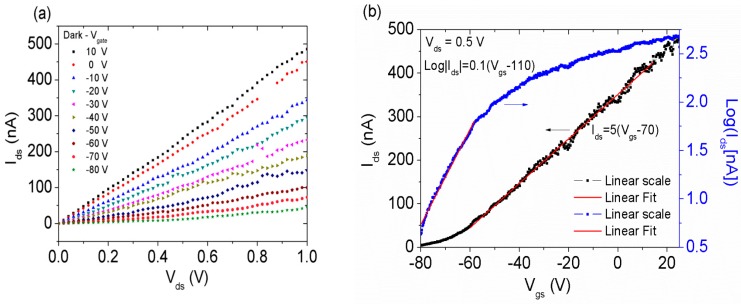
(**a**) Output characteristics $I_{\mathrm{ds}}-V_{\mathrm{ds}}$ of the ${\mathrm{MoS}}_{2}$ transistor for different values of the gate bias $V_{\mathrm{gs}}$; (**b**) Transfer characteristic $I_{\mathrm{ds}}-V_{\mathrm{gs}}$ (left scale) and $\mathrm{Log}|I_{\mathrm{ds}}|-V_{\mathrm{gs}}$ (right scale) with linear fittings.

Figure 2 reports the electrical transport characterization of the back-gate ${\mathrm{MoS}}_{2}$ transistor. The output characteristics $I_{\mathrm{ds}}-V_{\mathrm{ds}}$ (Figure 2a) were measured from −80 V to 10 V at gate voltage steps of 10 V. A channel resistance decrease, resulting in higher current, is observed for gate voltage, $V_{\mathrm{gs}}$, varying from negative to positive voltages. This is further evidenced by the transfer $I_{\mathrm{ds}}-V_{\mathrm{gs}}$ characteristics, shown in Figure 2b for a given drain–source bias voltage ($V_{\mathrm{ds}}=0.5\;V$).

The transfer curve discloses an n-type behavior with normally on channel at $V_{\mathrm{gs}}=0\;V$. The off-state of the transistor is reached below the negative gate voltage of −80 V that we safely adopted as the lower limit for $V_{\mathrm{gs}}$ to prevent ${\mathrm{SiO}}_{2}$ gate dielectric leakage or breakdown. The limited range of $V_{\mathrm{gs}}$ results in the apparent low on/off ratio of the transistor, which is essentially not turned off over the sweeping interval. Nevertheless, the measured portion of transfer curve is enough to estimate the threshold voltage. Given that $I_{\mathrm{ds}}\propto \left(V_{\mathrm{gs}}-V_{\mathrm{th}}\right)$, the threshold voltage corresponds to the x-axis intercept of the straight-line fitting of the current in linear scale and results in $V_{\mathrm{th}}\approx -70\;V$. We calculate the subthreshold swing as $\mathrm{SS}={\mathrm{dV}}_{\mathrm{gs}}/d\left({\mathrm{logI}}_{\mathrm{ds}}\right)$ = 20 V/decade using the transfer blue curve. The high value of SS, which is likely overestimated due to the fitting region being too close to $V_{\mathrm{th}}$, is expected because of the low-efficiency back-gate configuration with thick gate oxide.

Remarkably, the prevailing on-state over a wide $V_{\mathrm{gs}}$ range and the n-type doping suggest that the ${\mathrm{MoS}}_{2}$ flake can be suitable for electron extraction, i.e., for field emission applications.

We also evaluate the field-effect mobility from the slope of the transfer characteristic (black curve) via the following formula:

(1) $$\mu =\frac{{\mathrm{dI}}_{\mathrm{ds}}}{{\mathrm{dV}}_{\mathrm{gs}}}\frac{L}{W\cdot C_{\mathrm{ox}}\cdot V_{\mathrm{ds}}}$$

where $C_{\mathrm{ox}}=\varepsilon_{\mathrm{ox}}/d_{\mathrm{ox}}$ is the oxide capacitance; and $\varepsilon_{\mathrm{ox}}$ and $d_{\mathrm{ox}}$ are the ${\mathrm{SiO}}_{2}$ permittivity and thickness, respectively. For $300{\;\mathrm{nm}\;\mathrm{SiO}}_{2},{\;C}_{\mathrm{ox}}=11\;{\mathrm{nFcm}}^{-2}$ [45]. The obtained mobility, $0.046\;{\mathrm{cm}}^{2}V^{-1}s^{-1}$, is on the low side of the range typically reported for uncovered ${\mathrm{MoS}}_{2}$—$0.05\;{\mathrm{cm}}^{2}V^{-1}s^{-1}$ to $100\;{\mathrm{cm}}^{2}V^{-1}s^{-1}$ [8,33,46,47]. Our value for the field-effect mobility could be slightly underestimated because it does not exclude the effect of the contact resistances [48], which increase the total resistance of the sample and the probability of electron scattering. The low mobility is caused by the mentioned process residues, the long exposure to air, and the likely presence of defects in crystal structure.

### 3.2. Field Emission Measurements

**Figure 3:**
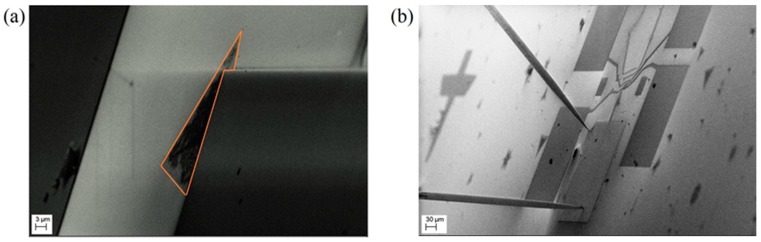
(**a**) SEM image of a ${\mathrm{MoS}}_{2}$ flake used for field emission measurements; (**b**) SEM image showing the positioning of the tungsten tips: the lower W -tip is on the metal pad contacting the flake, the other one is placed in front of the flake at a close distance d.

FE experimentation on a selected flake, shown in Figure 3a, was performed inside the SEM chamber by placing one of the two available W-tips on the metal electrode contacting the flake (cathode) and positioning the other tip (anode) at a variable distance d from the flake, as displayed in Figure 3b. 

Considering that the ${\mathrm{MoS}}_{2}$ flake is n-doped and that the sharp edge originates a high electric field amplification, we expect easy extraction of electrons from the edge of the flake upon application of a voltage. Nevertheless, exposure to air, flake oxidation, and the higher concentration of process residues at the edge make it harder to extract electrons from the flake boundary. On the other hand, due to a better surface quality (less contaminants) in the inner flat part of the flake, and taking advantage of the fine positioning control of our W-tip, we performed field emission characterization supported by SEM imaging from an internal, flat portion of the flake. FE from the flat part of ${\mathrm{MoS}}_{2}$ bilayers has not yet been reported in literature and constitutes an interesting way to complete the ongoing investigation of the field emission properties of ${\mathrm{MoS}}_{2}$. Similarly, FE measurements on graphene initially gave indication that emitting current was achievable only from edges [49], but it was later demonstrated that FE currents could be extracted from the inner flat part of a graphene flake under application of an electric field of a few hundred V/μm [50].

**Figure 4:**
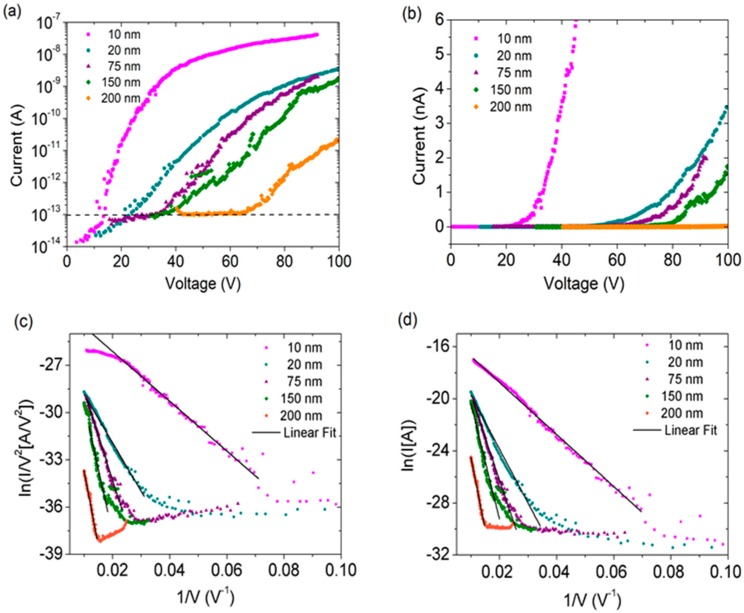
(**a**) Field emission current plotted as a function of applied voltage in semilogarithmic scale; the black line identifies the current level at which the turn-on field is defined; (**b**) Field emission current plotted as a function of applied voltage in linear scale; (**c**) Experimental data plotted with 3D Fowler–Nordheim model; (**d**) Experimental data plotted with modified 2D Fowler–Nordheim model, showing good fit over a wider range.

In Figure 4a,b, we show I−V curves in semilogarithmic and linear scale, respectively, at different anode–cathode distances (i.e., at variable separation between the W-tip and ${\mathrm{MoS}}_{2}$ surface). The curves show the typical fluctuations of emission current, indicating desorption of physisorbed molecules caused by Joule heating. Figure 4a shows that reducing the inter-electrode distance causes the field emission current to appear at lower voltages, confirming that the FE turn-on voltage depends on the electrode separation.

For a given distance, the current remains at the floor noise up to a threshold voltage, corresponding to what we define as the FE turn-on voltage; above this threshold it starts rising exponentially up to 100 nA, as expected from Fowler–Nordheim (FN) theory. According to FN model,

(2) $$I=\frac{{\mathrm{Sa}\beta }^{2}E^{2}}{\Phi }\exp\left[-\frac{{b\Phi }^{\frac{3}{2}}}{\beta E}\right]$$

where a and b are constants with values $1.54\times 10^{-6}\;A\;\mathrm{eV}\;V^{-2}$ and $6.83\times 10^{7}\;{\mathrm{eV}}^{\frac{3}{2}}\;V\;{\mathrm{cm}}^{-1}$, respectively; S and ϕ represent the emitting surface and the material work function (in our case ϕ=5.25 eV [51]); and β is the so-called field enhancement factor. β is a typical figure of merit for the qualification of field emitting materials, despite the fact that it has been shown to depend on the experimental setup [24]. Finally, the electric field is E=V/d, where V is the anode–cathode voltage.

The Fowler–Nordheim behavior of the field emission current is usually checked by the linearity of the so-called FN plot of $\ln\left(I/V^{2}\right)$ vs. 1/V. In particular, the intercept and slope of the fitting straight line yield the emission area and the field enhancement factor β, respectively. 

**Figure 5:**
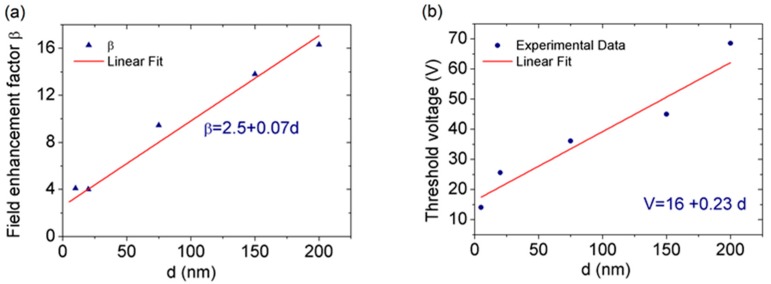
(**a**) Field enhancement factor as a function of the cathode–anode distance; (**b**) Threshold voltage as a function of cathode–anode distance.

The FN plot of our measurements is reported in Figure 4c, which confirms the expected linear behavior. Figure 4d is analogous to the FN plot for a modified Fowler–Nordheim model recently proposed by Yee Sin Ang et al. [42] to account for 2D electron confinement. This 2D FN model takes into account the fact that, differently from bulk materials, field emission from 2D materials may depend on the extraction direction, resulting in the following current field equation:

(3) $$I_{\mathrm{FN}}^{2D}=A_{\mathrm{FN}}^{2D}\exp\left[-\frac{{b\Phi }^{\frac{3}{2}}}{\beta E}\right]$$

where $A_{\mathrm{FN}}^{2D}$ is a constant, and the other symbols are the same as in Equation (2). Figure 4c,d show that both models well reproduce the experimental data. However, the 2D model provides a better fit over a wider voltage range. Thus, we use the 2D FN model for further analysis—in particular, to evaluate β at different distances d. The plot in Figure 5a shows linear behavior of β(d) in agreement with what has often been reported for different field emission sources [23,33,52,53]. Such behavior is likely due the fact that at higher distance the field from the W-tip becomes more uniform on the emitting area [21]. 

The seemingly low value of the amplification factor—less than 20 for d<200 nm—is remarkable if we consider that emission happens from the inner part of the flake, where no field enhancement by edge effect takes place, and that β further increases with the distance d. 

Finally, we can evaluate the turn-on field from the voltage values at which the current emerges from the noise floor of $1\times 10^{-13}A$. From the slope of the fitting straight line of the threshold voltage versus d plot, shown in Figure 5b, we estimate the turn-on field as 230 V/μm. If compared to the typical turn-on field of several kV/μm needed to extract electrons from flat surfaces, the obtained turn-on field can be considered a good result, pointing to noteworthy, although still unexploited, FE capabilities of ${\mathrm{MoS}}_{2}$. Like graphene, ${\mathrm{MoS}}_{2}$ is a flexible material [54]. The application of an electric field can cause local warpage of the flake and facilitate FE. Furthermore, surface roughness of the ${\mathrm{MoS}}_{2}$ flakes, which strongly depends on the substrate [55], creates wrinkles and protrusions which are favorable to FE. However, the main reason for the lower turn-on field is the n-doping and the low electron affinity of ${\mathrm{MoS}}_{2}$, which ranges from 3.74 eV to 4.45 eV [56,57,58] and is lower than that of graphene.

## 4. Conclusions

We have presented the electrical transport characterization of field-effect transistors with ${\mathrm{MoS}}_{2}$ bilayer channels. The conductance shows an n-type behavior and gate modulation, with prevailing on-state over a wide voltage range. This feature has suggested the use of ${\mathrm{MoS}}_{2}$ flakes for field emission investigations. We have reported significant field emission from the flat part of the flake under the application of a moderate electric field, even without taking advantage of field enhancement due to edge effects. We have also demonstrated that a modified field emission model, which considers the 2D nature of the flakes, provides a better fit compared with traditional 3D Fowler–Nordheim theory. This study, demonstrating the suitability of ${\mathrm{MoS}}_{2}$ as a field emitter, is a step ahead towards the exploitation of ${\mathrm{MoS}}_{2}$ for vacuum electronics applications, in addition to its established use as an electrode in heterojunctions and channel in field-effect transistors.
